# Theory of Electron Beam Moiré

**DOI:** 10.6028/jres.101.007

**Published:** 1996

**Authors:** David T. Read, James W. Dally

**Affiliations:** National Institute of Standards and Technology, Boulder, CO 80303; Mechanical Engineering Department, University of Maryland, College Park, MD 20742

**Keywords:** contrast, division, experimental mechanics, fringe, multiplication, pitch, rotation, spatial frequency, stress analysis

## Abstract

When a specimen surface carrying a high-frequency line grating is examined under a scanning electron microscope (SEM), moiré fringes are observed at several different magnifications. The fringes are characterized by their spatial frequency, orientation, and contrast. These features of the moiré pattern depend on the spatial frequency mismatch between the specimen grating and the raster scan lines, the diameter of the electron beam, and the detailed topography of the lines on the specimen.

A mathematical model of e-beam moiré is developed that expresses the spatial dependence of the SEM image brightness as a product of the local intensity of the scanning beam and the local scattering function from the specimen grating. Equations are derived that give the spatial frequency of the moiré fringes as functions of the microscope settings and the spatial frequency of the specimen grating. The model also describes the contrast of several different types of moiré fringes that are observed at different magnifications. We analyze the formation of these different fringe patterns, and divide them into different categories including natural fringes, fringes of multiplication, fringes of division, and fringes of rotation.

## 1. Introduction

When a specimen surface that carries a regular array of lines is examined under a scanning electron microscope (SEM), moiré fringes can be observed at several different magnifications. Some confusion can arise in the interpretation of the different fringe patterns, because the spatial frequency of the moiré fringes changes with mismatch, rotation, a multiplication phenomena, and a division phenomena. In this paper we first demonstrate these different fringe patterns, and then explain their formation based on a Fourier series representation.

Optical moiré fringes, either geometric or interferometric, are widely employed in experimental mechanics. The classical treatments of geometric moiré by Parks [1], Durelli and Parks [2], and Theocaris [3], and the descriptions of interferometric moiré by Post [4], Graham [5], and McKelvie [6] are most helpful in interpreting fringe pattern formation in e-beam moiré. However, certain features of the phenomenon of electron beam moiré were not anticipated in these classic treatments of optical moiré. These features result from the fact that in electron beam moiré, no actual reference grating exists. Instead, the electron beam raster scan replaces the reference grating.

The e-beam raster scan is similar in may respects to the video raster scan employed by Morimoto [7] in forming moiré fringes using low frequency specimen gratings. Kishimoto [8] recognized the similarity between the video and SEM raster scans and was the first to report the use of e-beam moiré fringes for experimental mechanics. However, neither Morimoto or Kishimoto discussed the many fringe patterns that may be observed when scanning lines are employed as the reference grating. With the controls available on a typical SEM it is possible to vary the e-beam diameter, the pitch of the raster scan, and the angle between the scan lines and the grating lines. All affect the fringe pattern.

We develop a mathematical model of e-beam moiré fringe formation that allows us to reproduce and extend certain results previously derived for optical moiré. The model is based on two postulates used in treatments of optical moiré [5]:

1. The spatial dependence of the pattern of the scan lines, and the spatial dependence of specimen grating, can be described using Fourier series.
2. The SEM image can be represented numerically as a set of intensity values given by the product of the scattering power of the specimen grating and the intensity of the scanning lines. Spatially extended interaction of the beam with the near-surface region of the specimen is incorporated as a contribution to the width of the scanning lines.

Based on these postulates, a model is derived that concisely describes natural moiré fringes, fringes of multiplication, and fringes of division. Experimental examples are demonstrated. The model is well-suited to determine the fringe contrast and the fringe shape as functions of the raster scan pitch, the scan line width, and specimen grating parameters.

## 2. Observation of Specimen Gratings and e-beam Moiré Fringes

**Fig. 1:**
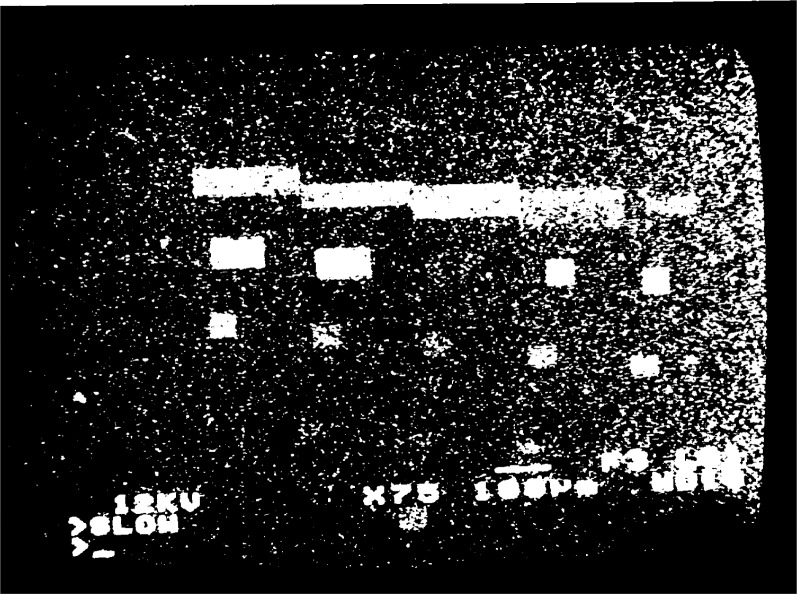
Several line gratings written with different frequencies and exposures, on a brass specimen.

Several high-density gratings, with spatial frequencies $f_{g}^{\prime }$ of 2.5 μm^−1^ to 10 μm^−1^, were written on a brass specimen using the methods described in [9]. A macroscopic view of the small areas written with different frequencies and different e-beam exposures is presented in Fig. 1. Examination of a grating with $f_{g}^{\prime }=5\mu m^{-1}$ at high magnification, Fig. 2, shows the appearance of the grating lines on the specimen. Depending on the effectiveness of the process used to fabricate such lines, they may appear in the SEM display as high-contrast stripes of black and white, as shown in Fig. 2, or as low contrast stripes represented by intensity modulations in a gray field. Local imperfections in the specimen surface and in the grating produce irregularities in the brightness of the image. Additional imperfections are generated by the imaging process, even though the SEM image is recorded at a slow scan rate.

When a grating with $f_{g}^{\prime }=5\mu m^{-1}$ is observed, moiré fringes appear at several different magnifications over the range 300 to 3000. Typical moiré patterns are illustrated in Figs. 3 to 5. We have divided these fringe patterns into three categories, based on the relative sizes of $f_{g}^{\prime }$, the spatial frequency of the specimen grating, and *f*_b_, the spatial frequency of the raster scan. Moiré fringes of division, where $f_{g}^{\prime }>f_{b}$, are presented in Figs. 3a and 3b. Natural moiré fringes where $f_{g}^{\prime }$ and *f*_b_ are nearly equal are shown in Figs. 4a to 4e. Most of these fringe patterns represent a slight mismatch between the pitch of the raster scan and the pitch of the grating; however, Fig. 4c represents nearly a perfect match. Moiré fringes of multiplication, first observed optically by Post [8], are also observed with e-beam moiré when $f_{b}>f_{g}^{\prime }$. Multiplication by two and three is illustrated in Figs. 5a and 5b.

## 3. Theory of e-beam Moiré Fringe Formation

We introduce a theory to describe the formation of the several different types of moiré fringes that are observed in an SEM. The theory is similar to that introduced to describe the formation of fringes in optical geometric moiré. Fourier series representations are used to describe the SEM raster scan, the specimen line grating, and the moiré fringes. The results are interpreted to explain the occurrence of fringes classified as natural, multiplied, and divided. The description of fringes of rotation is adapted directly from optical moiré.

### 3.1 The SEM Raster Scan System

The image observed in an SEM is produced by scanning the specimen grating with an e-beam raster scan. We locate a point in this image by its coordinates (*x*, *y*). The e-beam is scanned continuously across the imaged field in the *x* direction. The e-beam scan lines are equally spaced, with pitch *p*_b_ in the y direction. The magnified image, viewed on the CRT display, has a nominal size of 90 mm in the *y* direction. This dimension is related to a common photomicrograph size. The design of the SEM is such that the raster pattern is always aligned with the viewing screen and the camera frame, so in all the SEM images the *x*-axis is horizontal and the *y*-axis is vertical.

The specimen carries a line grating that consists of an array of lines extending in the ± *x*′ direction, spaced equally with pitch $p_{g}^{\prime }$ in the *y*′-direction. The reciprocal of $p_{g}^{\prime }$ is $f_{g}^{\prime }$, the spatial frequency of the specimen grating. The beam and specimen coordinates may be rotated with respect to one another by a control on the SEM. The angle between the *x* and *x*′ axes is *θ*.

The number of scan lines used to form the image can be set at various values. Typical nominal settings are 500, 1000, or 2000 scans to produce an image. The images in Figs. 3 to 5 were made with 500 lines. Possible magnification values range from 10 to 300 000. Because of the design of our microscope, only certain discrete values of the magnification are available. As a consequence, it is very unusual for us to achieve a null-field moiré fringe pattern.

The pitch of the electron beam raster scan lines, *p*_b_, depends on the magnification, *M*, the nominal image size, *S*, and the number of raster scans used to make the image, *R*, as

$$p_{b}=S/MR.$$ (1)

For example, with 500 lines per image, a nominal image height of 90 mm, and a magnification of 1900, the scan pitch *p*_b_ is 95 nm.

The effective width of the electron beam scan lines depends on the actual e-beam diameter and the interaction of the beam with the specimen surface. Beam diameters of 5 nm to 20 nm are reported in the literature and in the specifications for our SEM. Attainment of very small beam diameters (10 nm) requires very low beam currents, a well-aligned microscope, a small aperture, and extremely sharp focussing. The interaction zone diameter depends on the specimen material and the electron beam energy (accelerating voltage). We believe a value of 15 nm to 30 nm is typical for the effective width of the raster scan lines used in this study.

The specimen gratings are formed by etching thin troughs in a polymeric film about 100 nm thick. The frequencies obtained vary from 2.5 μm^−1^ to 10 μm^−1^. The lines (troughs) appear as dark stripes in the image and the ridges between the troughs appear as light stripes. In our densest gratings, the width of the troughs and the ridges is approximately equal. A *y*′-direction trace of the image intensity shows a profile with gradual, rather than abrupt, changes in the image intensity.

### 3.2 Fourier Representations of the Grating and Scanning Lines

We follow the approach introduced by Sciamarella [8] for optical moiré; we assume that the local intensity (brightness) of the image is proportional to the product of the local scattering power of the specimen grating and the local intensity of the e-beam scan line. The scattering function *G*(*y*′) for the specimen grating is represented by a Fourier series:

$$G(y^{\prime })=\frac{g_{0}}{2}+\sum_{n=1}^{\infty }g_{n}\cos\;(2\pi nf_{g}^{\prime }y^{\prime })$$ (2)

where the *g_n_* are Fourier coefficients and $f_{g}^{\prime }$ is the spatial frequency of the grating lines. After deformation, the specimen grating frequency $f_{g}^{\prime }$ can vary with position over the specimen. However, in this treatment we simplify the analysis by considering only deformation fields that produce constant strain over the local region of interest. The frequency $f_{g}^{\prime }$ represents the current value at the time of image formation, which is usually different from the original value.

The intensity of the e-beam scan lines *B*(*y*) is also represented by a Fourier series:

$$B(y)=\frac{b_{0}}{2}+\sum_{m=1}^{\infty }b_{m}\cos\;(2\pi mf_{b}y)$$ (3)

where the *b_m_* are Fourier coefficients and *f*_b_ is the spatial frequency of the raster lines. In both Eqs. (2) and (3), the cosine representation is sufficiently general because somewhere in the image an origin can be found such that the sine terms vanish.

In the simplest case, the *y* and *y*′ axes coincide. But the raster scan lines can be rotated at an angle *θ* relative to the grating axes to produce angular misalignment. When *θ* ≠ 0, we will transform *G*(*y*′) into the coordinates of the raster scan and the image. Since *G*(*y*′) is a periodic function *y*′ with no dependence on *x*′, the Fourier representation is valid in the transformed coordinates. However, for *θ* ≠ 0 the dependence of the grating scattering function on *x*′ cannot be ignored, and we will consider the specimen grating to be represented by *G*(*x*′, *y*′). It is convenient to measure the moiré fringe spatial frequency *f*_i_ along the *y*-direction, which is vertical in the SEM images. The spatial frequency of the e-beam raster scan pattern is naturally measured along the *y*-direction. It is convenient to have all spatial frequencies referred to the same axis, so for calculations we transform the value of the grating frequency into a new value, *f*_g_, the effective grating frequency in the raster scan coordinate system. We take $f_{g}=f_{g}^{\prime }\cos(\theta )=\cos(\theta )/p_{g}^{\prime }$, where $p_{g}^{\prime }$ is the physical grating pitch, measured in the *y*′ direction. In practice, *θ* is usually adjusted to be 0 when moiré fringe patterns are being recorded, so in such cases $f_{g}=f_{g}^{\prime }=1/p_{g}^{\prime }$.

The moiré pattern intensity *I*(*x*, *y*) is represented as the product of the raster function and the grating function:

I(y)=B(y)G(y). (4)

Substituting Eqs. (2) and (3) into Eq. (4) and arranging the products of the cosine terms into sum and difference cosine functions gives a relation of the form

I(y)=C+F(y)+S(y)+D(y), (5)

where *C* = *g*_0_*b*_0_/4 is a constant. The function

$$\begin{array}{c} F(y)=\left(\frac{b_{0}}{2}\right)\sum_{n=1}^{\infty }g_{n}\;\cos\;(2\pi nf_{g}y)\\ +\left(\frac{g_{0}}{2}\right)\sum_{m=1}^{\infty }b_{m}\;\cos\;(2\pi mf_{b}y) \end{array}$$

exhibits a frequency that is too high to be observed. The sum function

$$S(y)=\sum_{m=1}^{\infty }\sum_{n=1}^{\infty }\left(\frac{g_{n}b_{m}}{2}\right)\cos\;2\pi (nf_{g}+mf_{b})y$$

also exhibits a frequency that is too high to be observed. The difference function

$$D(y)=\sum_{m=1}^{\infty }\sum_{n=1}^{\infty }\left(\frac{g_{n}b_{m}}{2}\right)\cos\;2\pi (nf_{g}-mf_{b})y$$

is the term in the double series expansion that produces the image observed and identified as the moiré fringe pattern.

We simplify Eq. (5) to give

$$I(y)=C_{1}+\sum_{m=1}^{\infty }\sum_{n=1}^{\infty }\left(\frac{g_{n}b_{m}}{2}\right)\cos\;2\pi (nf_{g}+mf_{b})y$$ (6)

where *C*_1_ = *C* + *F*(*y*) + *S*(*y*) is the intensity of the background.

The result is similar to that obtained in optical moiré. When the magnification yields moiré fringes, neither the grating lines nor the scan lines can be clearly imaged.

The coefficients *g_n_* in the specimen grating function *G*(*y*′) fall off rapidly with n because of the topography of the grating. The coefficients *b_m_* of the scanning beam raster function *B*(*y*) do not decay as rapidly with increasing order *m* of the expansion. The reason for the persistence in *b_m_* is described later.

### 3.3 Natural Moiré Fringes

The simplest condition for fringe formation in optical moiré is when *f*_g_ is approximately equal to *f*_b_; this is the near-match condition. Similarly in e-beam moiré we refer to fringes formed under this near-match condition as natural fringes. Because only discrete values of magnification are available on our SEM, we have never been able to achieve a perfect null field, where *f*_g_ = *f*_b_ and the pitch of the moiré fringes *p*_m_ becomes infinite.

Considering the first term in the sum in Eq. (6) (*n* = *m* = 1) for the near match condition where *f*_b_ ≈ *f*_g_ gives the frequency *f*_i_ of the moiré fringe intensity function *I*(*y*):

$$f_{i}=f_{g}-f_{b}.$$ (7)

In Eq. (7), negative values of the moiré fringe frequency are allowed, because moiré fringes are formed both for *f*_g_ > *f*_b_ and for *f*_g_ < *f*_b_. Because the cosine is a symmetric function of its argument, it is impossible to determine from Eq. (6) whether *f*_b_ or *f*_g_ is greater. This is important in measurements, because it represents the difference between expansion and contraction of the specimen. In practice this ambiguity is resolved by changing the magnification which in turn changes *f*_b_ in a known sense. The result is a change in *f*_i_ that can be observed and interpreted to determine if *f*_b_ is higher or lower than *f*_g_.

Consider small uniform longitudinal strains along the *y* direction, relative to the ideal null condition where *f*_g_ = *f*_b_ and *θ* = 0. Equation 7 implies that the tensile strain *ε* is given by

$$\varepsilon =-\frac{f_{i}}{\left(f_{b}+f_{i}\right)}.$$ (8)

The periodic form of Eqs. (2) to (6) makes it possible to adopt a vast body of previous developments to interpret e-beam moiré fringes. Some familiar wave phenomena have analogs in SEM images of line gratings. For example, it is clear from Eq. (7) that the moiré fringes are analogous to the beat frequency due to two pure sound tones of slightly different frequencies. Another example is the Doppler phenomena. We observed a changing frequency of the moiré fringe pattern when the specimen was moved under the scanning electron beam.

The contrast of the natural moiré fringes is determined primarily by the amplitude term *g*_1_*b*_1_/2, although higher order terms also affect the contrast. Higher order harmonics of the fringe frequency occur for *m* = *n* = 2, 3, … etc. These harmonics distort the pure sinusoid of the fundamental. Other higher order terms that occur when *n* ≠ *m* produce signals with a very high frequency that can be disregarded except for their detrimental effect on contrast. Post [9] has used specially selected aspect ratios between bar and space widths in optical moiré to produce “fringe sharpening” effects. Equation (6) shows that the effect of bar and space widths on moiré fringe shape can be calculated by Fourier series techniques. This phenomenon is qualitatively observable in e-beam moiré as a resemblance between the appearances of the grating lines at high magnification and the moiré fringes at low magnification.

### 3.4 Fringes of Multiplication

Post [11] showed that fringe multiplication occurred in optical moiré when the spatial frequency of the reference grating was a near multiple of the spatial frequency of the specimen grating. The same fringe multiplication occurs in e-beam moiré. We express the spatial frequency of the scan lines, following the notation introduced by Post [11] as:

$$f_{b}=\beta (1+\lambda )f_{g}$$ (9)

where *β* is a positive integer and *λ* is a small fraction. Substituting Eq. (9) into Eq. (7) shows that the spatial frequency of the moiré fringe pattern intensity is:

$$f_{i}=[n-m\beta (1+\lambda )]\;f_{g}=[(n-\beta m)-m\lambda \beta ]f_{g}.$$ (10)

Moiré fringes may be observed when *n* = *β m*; then *f*_i_ becomes

$$f_{i}=-m\lambda \beta f_{g}.$$ (11)

The amplitude of the moiré fringe terms in Eq. (6) is given by *g_n_b_m_*/2. Since *β* is typically an integer from 2 to 5, *n* = *βm* is always greater than one, while maximum contrast requires *m* be fixed at 1. This fact means that fringes of multiplication occur when we match the fundamental frequency of e-beam raster scan with the second, third, etc., harmonics of the grating function. Difficulties in obtaining high contrast fringes of multiplication are due to the decreasing Fourier amplitudes of higher harmonics of the grating function. To illustrate this important result, let *β* = 2 and consider a specimen grating that is represented by a symmetric square wave (an amplitude grating with bar width equal to space width). Since *g*_2_, the coefficient of the second harmonic for a square wave, is 0, the contrast goes to zero and these fringes of multiplication cannot be observed.

These results show the importance of the grating scattering function for the contrast of fringes of multiplication. A grating with narrow lines and wide spaces exhibits stronger even harmonics than a balanced grating with equally wide lines and spaces. However, for all shapes, except the periodic delta function, the general rule is that the coefficients *g_n_* decrease rapidly with increasing order of the harmonic. Unfortunately, we have not been able to produce high-density specimen lines that scatter like delta functions by e-beam lithography. Our highest-density gratings scatter much like sinusoids.

Our experience with fringes of multiplication is that they are difficult if not impossible to observe, as is consistent with the preceding development. Examples of fringe multiplication by two and three, Fig. 5, show fringes with lower contrast than those in Figs. 3 and 4.

### 3.5 Fringes of Division

Moiré fringes of division also occur, but in this case the specimen grating frequency is a multiple of the scan line frequency. Fringes of division are commonly observed at low magnification settings on the SEM, where *p*_b_ is larger than *p*_g_. The formation of the fringes of division and their contrast can be explained by using the Fourier representation. Consider an observation in the SEM with a frequency relation given by

$$f_{g}=\alpha (1+\gamma )f_{b}$$ (12)

where *α* is an integer and *γ* is a small fraction. The frequency of the resulting moiré fringe intensity is obtained from Eq. (7) as:

$$f_{i}=[(\alpha n-m)+\alpha \gamma n]f_{b}.$$ (13)

The moiré pattern can be observed when *αn* = *m* and Eq. (13) reduces to:

$$f_{i}=\alpha \gamma nf_{b}$$ (14)

Since α is an integer typically from 2 to 5, *m* = α*n* is always greater than one. This shows that moiré fringes of division are formed by combining the fundamental frequency component of the specimen grating with higher harmonics of the e-beam raster pattern. The e-beam scan lines that are produced at low magnification have relatively high coefficients *b_n_* for *n* as large as 10.

**Fig. 6:**
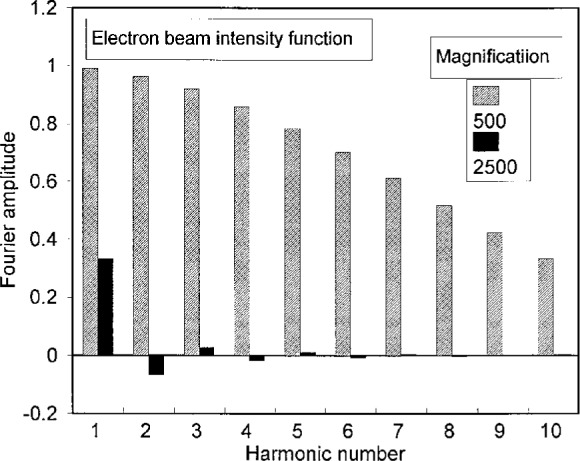
Estimated relative amplitudes of terms in the Fourier expansion of the electron beam raster scan function *B*(*y*), for magnifications of 500 and 2500.

To show the strength of the higher order harmonics associated with the e-beam scan lines, consider a magnification *M* = 500. With the number of raster lines *R* = 500, this setting on the SEM gives a pitch *p*_b_ = 360 nm by Eq. (1). This value of *p*_b_ is about 20 times larger than the effective width of the scan line (18 nm). Since the raster scan is only sampling about 5 % of the specimen surface, it behaves like a periodic series of delta functions in *y*. We show the relative magnitude of the Fourier coefficients for a scan line width *w*_b_ = 0.05 *p*_b_ in Fig. 6. It is evident that the coefficients *b_m_* decrease slowly with increasing *m*. For this reason fringes of division may be observed at many low magnifications with excellent contrast as illustrated by Fig. 3.

As the magnification is increased, *p*_b_ decreases and the portion of the field sampled by the scan lines increases. When the ratio *w*_b_/*p*_b_ increases, the coefficients of the higher harmonics of *B*(*y*) decrease more rapidly with order m. This phenomenon is illustrated in Fig. 6 where the coefficients are shown for *M* = 2500 and *w*_b_/*p*_b_ = 0.25.

### 3.6 Fringes of Rotation

**Fig. 7:**
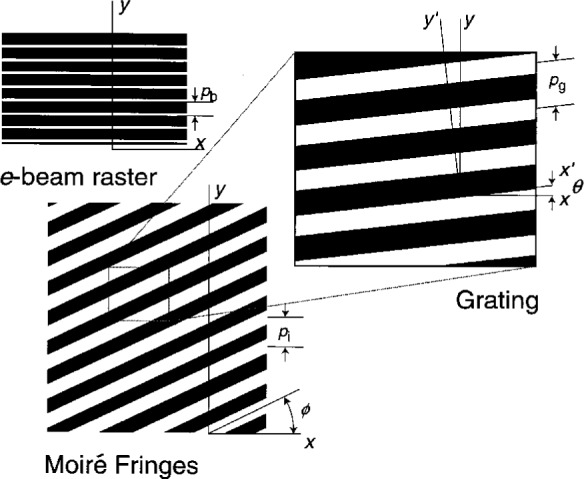
Definition of geometry and signs of angles when the specimen grating and the electron beam raster scan are not aligned.

Fringes of rotation occur in optical moiré when the pitch of the specimen and reference gratings are closely matched and one grating is rotated relative to the other [1]. Similar fringes of rotation also occur in e-beam moiré. We restate the relevant equation here because the phenomenon is quite commonly observed in e-beam moiré, and is useful. We consider the specimen grating lines to be along the *x*′ axis and the scan lines along the *x* axis. The angle measured from the *x* axis to the *x*′ axis is *θ*, which can be of either sign. When the rotation knob on the SEM is turned clockwise, the specimen’s image also appears to rotate clockwise. Of course, the specimen is not actually rotating. The raster scan lines are rotating in the opposite sense. From alignment at *θ* = 0, counterclockwise rotation of the SEM rotation control produces a clockwise rotation of the raster scan pattern, and thus a positive angle *θ*. We measure the fringe angle ϕ from the *x* axis. The moiré fringes of rotation make an angle of ϕ with the *x* axis, as shown in Fig. 7.

$$\tan\phi =\frac{\sin\theta }{\cos\theta -\frac{p_{g}}{p_{b}}}.$$ (15)

At match conditions where *p*_g_ = *p*_b_, Eq. (15) reduces to:

$$\phi =\frac{\pi }{2}+\frac{\theta }{2}.$$ (16)

Equation (16) indicates that at match for small *θ*, the moiré fringes are nearly perpendicular to both the grating lines and the raster scan lines. For typical mismatch but small misalignment where sin *θ* ≈ *θ*, Eq. (15) reduces to:

$$\phi \approx \theta \left(\frac{f_{g}}{f_{i}}\right).$$ (17)

Equation (17) shows that small misalignments produce much larger fringe angles, because *f*_g_/*f*_i_ is a large quantity whenever moiré fringes are visible. In Eq. (17), ϕ, *θ*, and *f*_i_ can be positive or negative, but *f*_g_ is always positive. The value of *θ* can easily be adjusted in the SEM by a control which rotates the direction of the raster scan pattern. This is helpful because Eq. (17) shows that the sense of the change of ϕ with *θ* gives the sign of *f*_i_.

## 4. Magnification, Field Size, and Mismatch

**Fig. 8:**
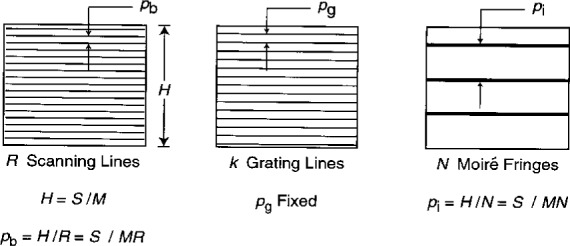
Illustrations of raster scan lines, grating lines and moiré fringes when the grating area exceeds the field of view.

We showed in Eq. (1) that the magnification *M* and the number *R* of lines in the SEM raster scan determine the pitch *p*_b_ of the scanning lines. As the settings on the SEM are changed, both the size of the field and the e-beam moiré fringe pattern change. A schematic illustration of the field size, the specimen grating, the scan line raster, and the moiré fringe pattern is presented in Fig. 8.

The field size or observation height *H* is given by:

$$H=\frac{S}{M}.$$ (18)

The pitch of the specimen grating is fixed at *p*_g_ for a given experiment. Here we assume that the specimen grating and the raster scan lines are perfectly aligned, so we do not need to differentiate between primed and unprimed coordinates. As long as the height of the grating, *h* exceeds the field size *H*, he number of grating lines in the field of observation, *k*, changes with *M* as:

$$k=\frac{S}{Mp_{g}}.$$ (19)

The number *N* of moiré fringes across the field of view is:

N=R−k (20)

and the pitch of the moiré fringes *p*_i_ is:

$$p_{i}=\frac{H}{N}=\frac{S}{MN}.$$ (21)

We have defined the fringe order *N* as positive when the number of specimen grating lines in the field of view exceeds the number of scan lines [(Eq. 7)]. With this definition, we admit both positive and negative fringe orders. By combining these equations, we can show the relation between the moiré fringe frequency and the SEM settings (*R* and *M*) as

$$f_{i}=\frac{1}{p_{i}}=\frac{1}{p_{g}}-\frac{RM}{S}.$$ (22)

Equation (22) derives from Eq. (7), which was *f*_i_ = *f*_g_ − *f*_b_. At high magnifications, the spatial frequency of the electron beam raster scan is higher than that of the specimen grating, so the frequency of the moiré fringe pattern f_i_ is negative. The conventional usage [2] is that *f*_i_, *p*_i_, and *N* are all positive by definition. However, this choice gives rise to the ± in many equations. To avoid this awkward notation, we allow *f*_i_, *p*_i_, and *N* to be either positive or negative so that the relevant equations are single-valued.

The number of fringes *N* observed in the field of view is:

$$N=Hf_{i}=H(f_{g}-f_{b})=H\left(f_{g}-\frac{MR}{S}\right)$$ (23)

when the grating height *h* > *H*, the field size. Otherwise

$$N=h\left(f_{g}-\frac{MR}{S}\right)$$ (24)

when the grating height *h* < *H*.

The match condition, which gives the null field, *f*_i_ = 0, is the same in both cases:

$$M=\frac{S}{R}\frac{1}{p_{g}}=\frac{S}{R}f_{g}.$$ (25)

For example, a specimen grating with *f*_g_ = 5 μm^−1^ observed in an SEM with *S* = 90 mm and *R* set at 500 will yield a null field associated with the natural match condition when *M* = 900. Match conditions for fringes of division and multiplication will occur at magnifications of 900/*α* or at 900 *β*, for *α* and *β* integers.

**Fig. 9:**
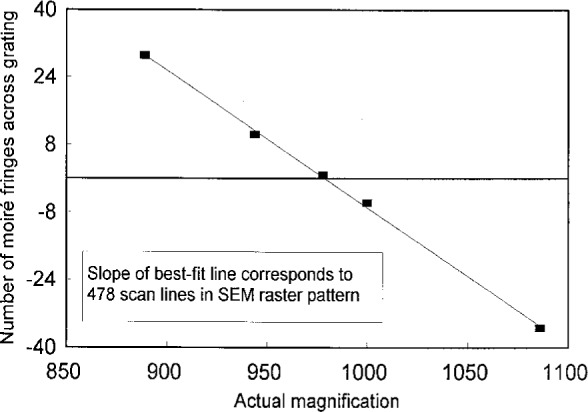
Fringe count *N* over specimen grating at different magnifications.

An experiment was conducted using a specimen grating with *f*_g_ = 5 μm^−1^. The grating area was small (*h* = 63 μm) so that *h* < *H* for all choices of *M* used in the experiment. The SEM was operated with nominal values of *R* = 500 and *S* = 90 mm. The magnification was changed so we observed mismatch conditions with both positive and negative fringes. The results for *N* as a function of *M*, presented in Fig. 9, show a linear relation as expected from Eq. (24). The slope of the *N* vs *M* relation is [from Eq. (24)]

$$\frac{dN}{dM}=\frac{-hR}{S}.$$ (26)

The slope was determined from the least squares fit of the data as −0.325. We also noted that the match condition occurred at *M* = 977. From the match condition we could determine that *S*/*R* = 195.4 μm. For *S* = 90 mm we find that *R* = 478 lines. This result for *R* agrees with the nominal value supplied by the manufacturer of the SEM.

## 5. Magnification Calibration

**Fig. 10:**
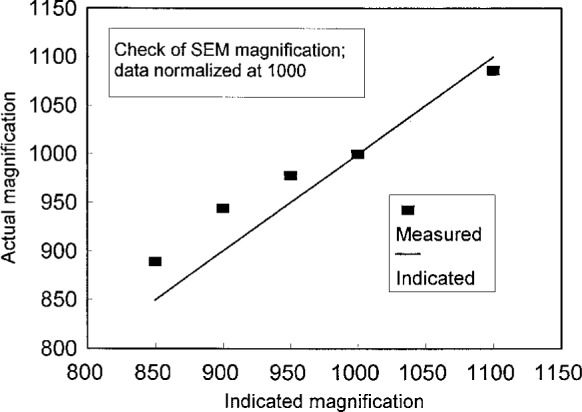
Actual versus indicated magnification showing relative errors in the SEM magnification calibration. Data normalized to a magnification of 1000.

The interpretation of moiré fringe patterns depends on precise knowledge of the magnification at any setting M employed to produce a pattern. To check the accuracy of the magnification of the SEM, we measured the length of a distinctive feature on the surface of a specimen at *M* = 1000. We then measured the length of this same feature at other magnifications as indicated on the SEM character display. We found that the actual magnifications were different from the indicated magnifications, as shown in Fig. 10. In preparing Fig. 10 we assumed that the microscope was absolutely correct at *M* = 1000. An improved calibration technique should employ a calibration standard so that the accuracy of all magnification settings could be established.

Examination of Fig. 10 shows that the magnification errors are less than 5 %. Nevertheless the errors are significant in the present analysis as accurate magnification values must be employed in Eq. (23) to properly characterize the scanning line function produced by the SEM. Similarly, the micrometer bar, which also appears in the image display of many microscopes, must also be accurately calibrated if it is to be useful in the quantitative interpretation of e-beam moiré fringes.

The actual magnifications given in Fig. 10 were employed in preparing the data presented in Fig. 9.

## 6. Conclusions

The formation of e-beam moiré fringes in a SEM can be described with a model based on a Fourier series representation of the specimen grating line function *G*(*y*′) and the raster scan line function *B*(*y*). The moiré fringe intensity function *I*(*y*) is the product of these two functions. The model describes the variation in the spatial frequency *f*_i_ of the moiré fringes with the magnification used in producing the image. It also provides a means for estimating the contrast of different moiré fringe patterns that are observed in the SEM. The spatial frequency *f*_i_ of the moiré fringes can be used to measure the spatial frequency *f*_g_ of the specimen grating to determine local displacements and strains.

The sensitivity and resolution of measurements made with e-beam moiré are limited by the frequency of the specimen grating. Fringes of multiplication offer enhanced displacement sensitivity per fringe, but require that the specimen grating be fabricated with a trough-ridge ratio that produces substantial higher order Fourier components. Fringes of division are observed as easily as natural moiré fringes because the raster scan lines at low magnifications exhibit significant Fourier coefficients for the higher order terms in the expansion. Fringes of division are useful because they permit a larger field of observation while maintaining the same displacement sensitivity per fringe as is achieved with the natural moiré patterns.

Fringes of rotation are easy to observe by operating the SEM control for the e-beam scan line direction. This control is useful for alignment and for establishing the sign of the moiré fringe frequency *f*_i_.

A method was described for characterizing the SEM based on determining mismatch fringes at different magnifications. The method is dependent on the use of accurate magnification values. We found that calibration of the SEM at each discrete magnification setting was essential.

## Figures and Tables

**Fig. 2a:**
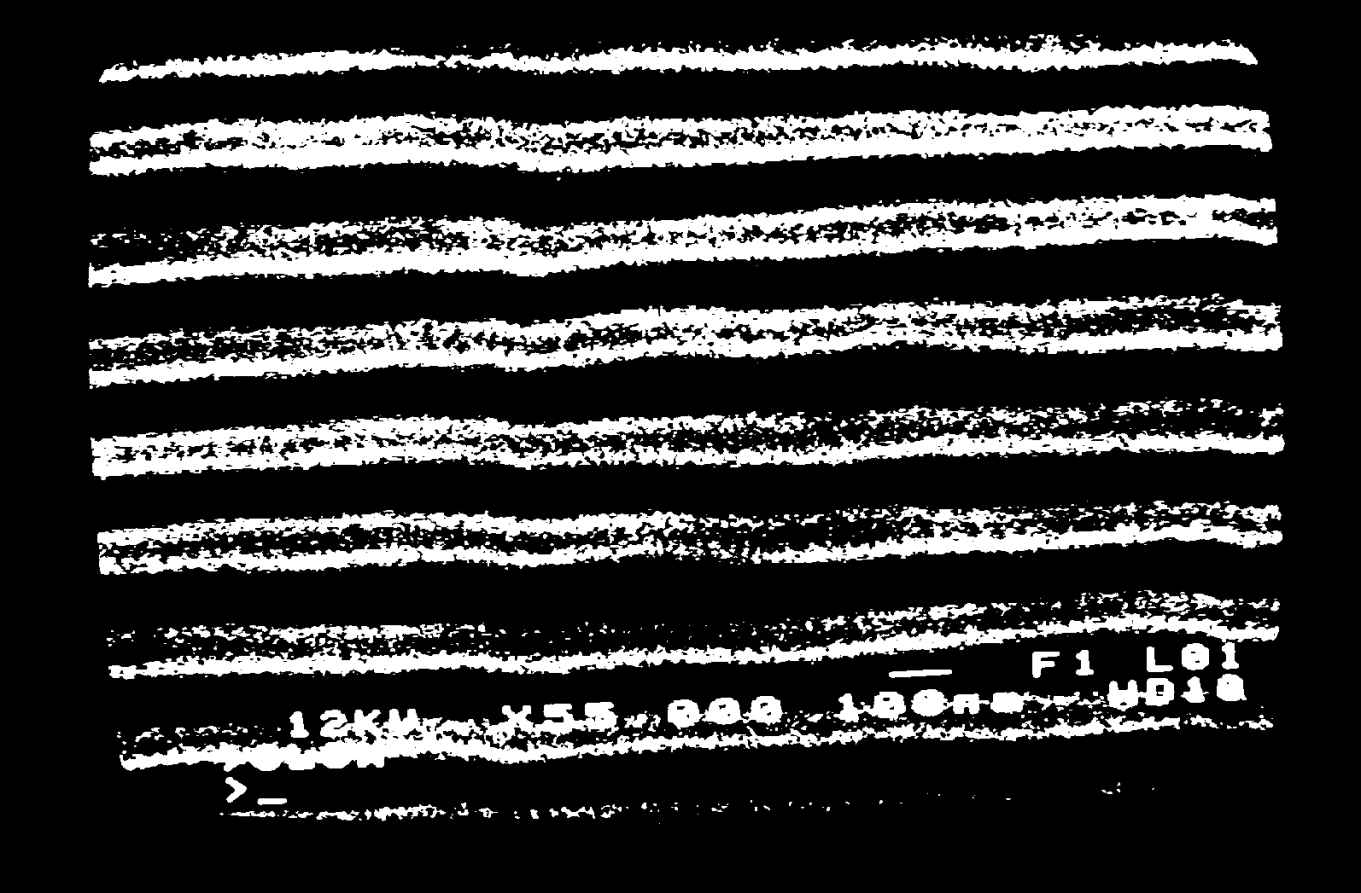
SEM image of a line grating with *p*_g_ = 220 nm, at a magnification of 55 000.

**Fig. 2b:**
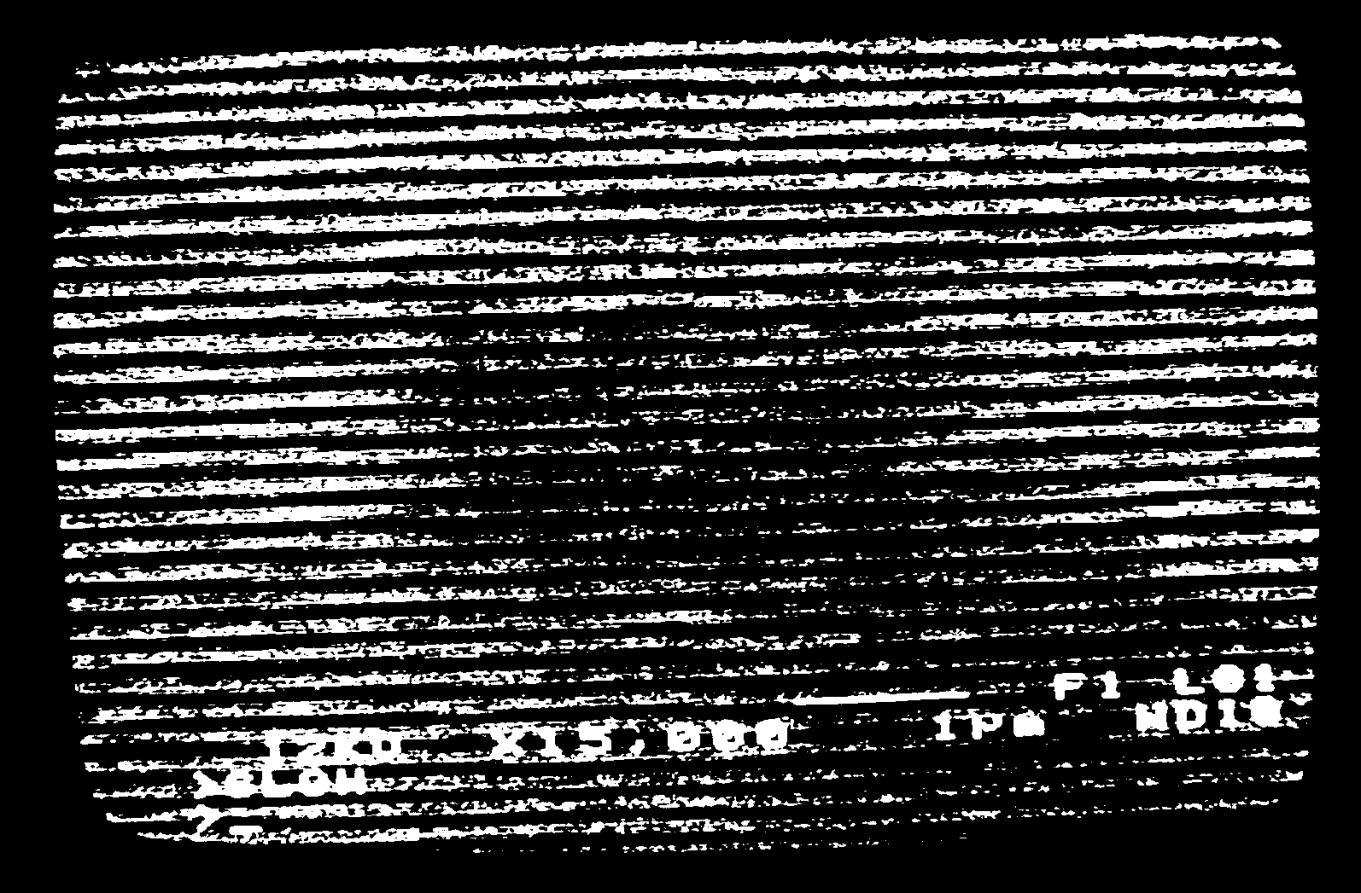
As in Fig. 2a, except at a magnification of 15 000.

**Fig. 3a:**
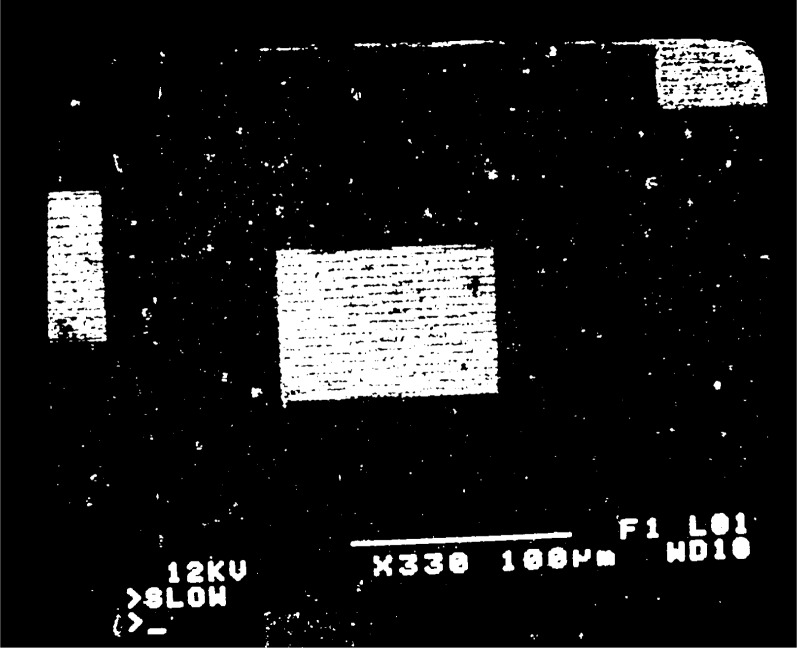
Moiré fringes of division on a 200 nm line grating, at a magnification of 330.

**Fig. 3b:**
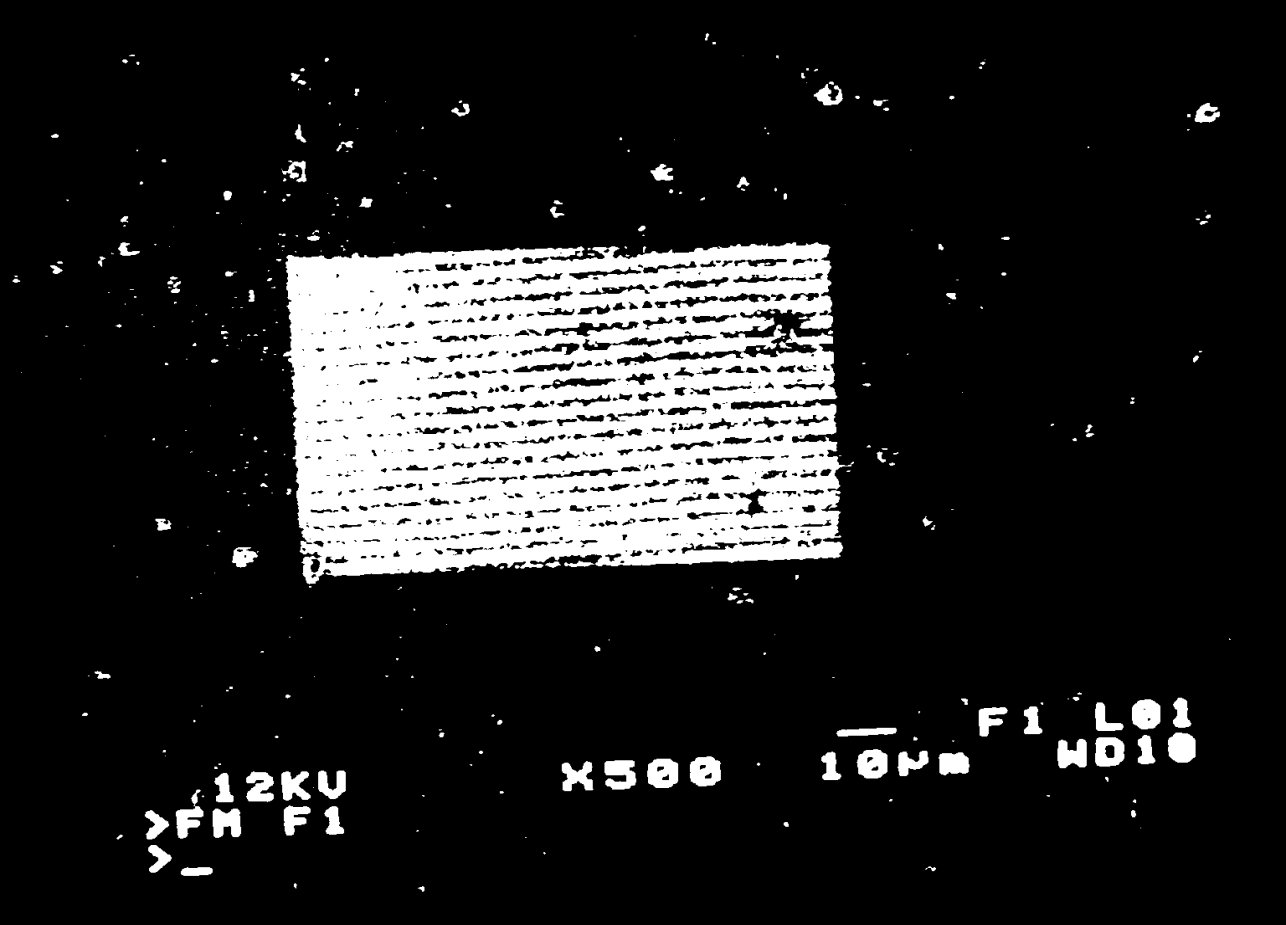
As in Fig. 3a, except at a magnification of 500.

**Fig. 4a:**
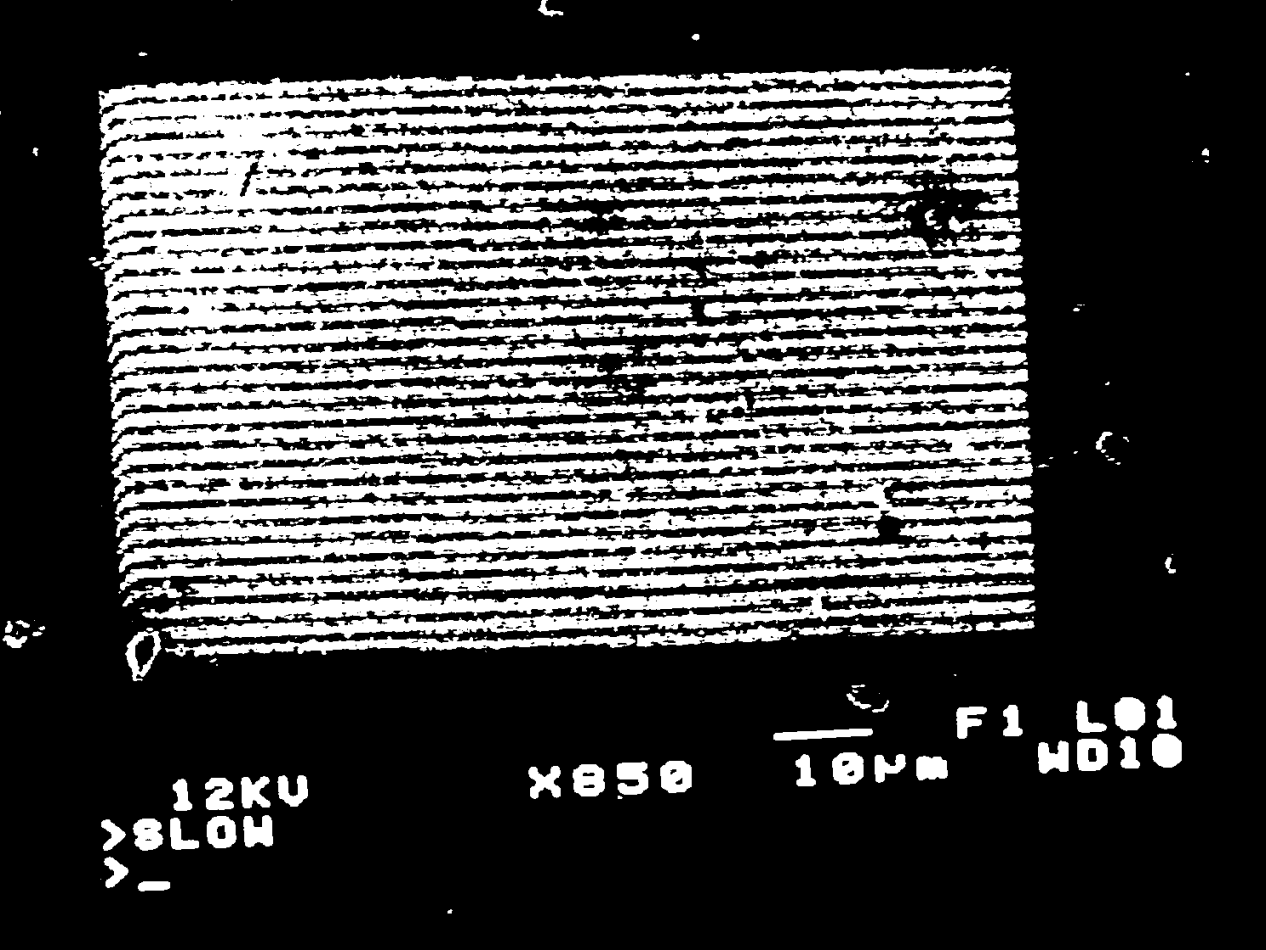
Natural moiré fringes with *p*_g_ = 200 nm, at a magnification of 850.

**Fig. 4b:**
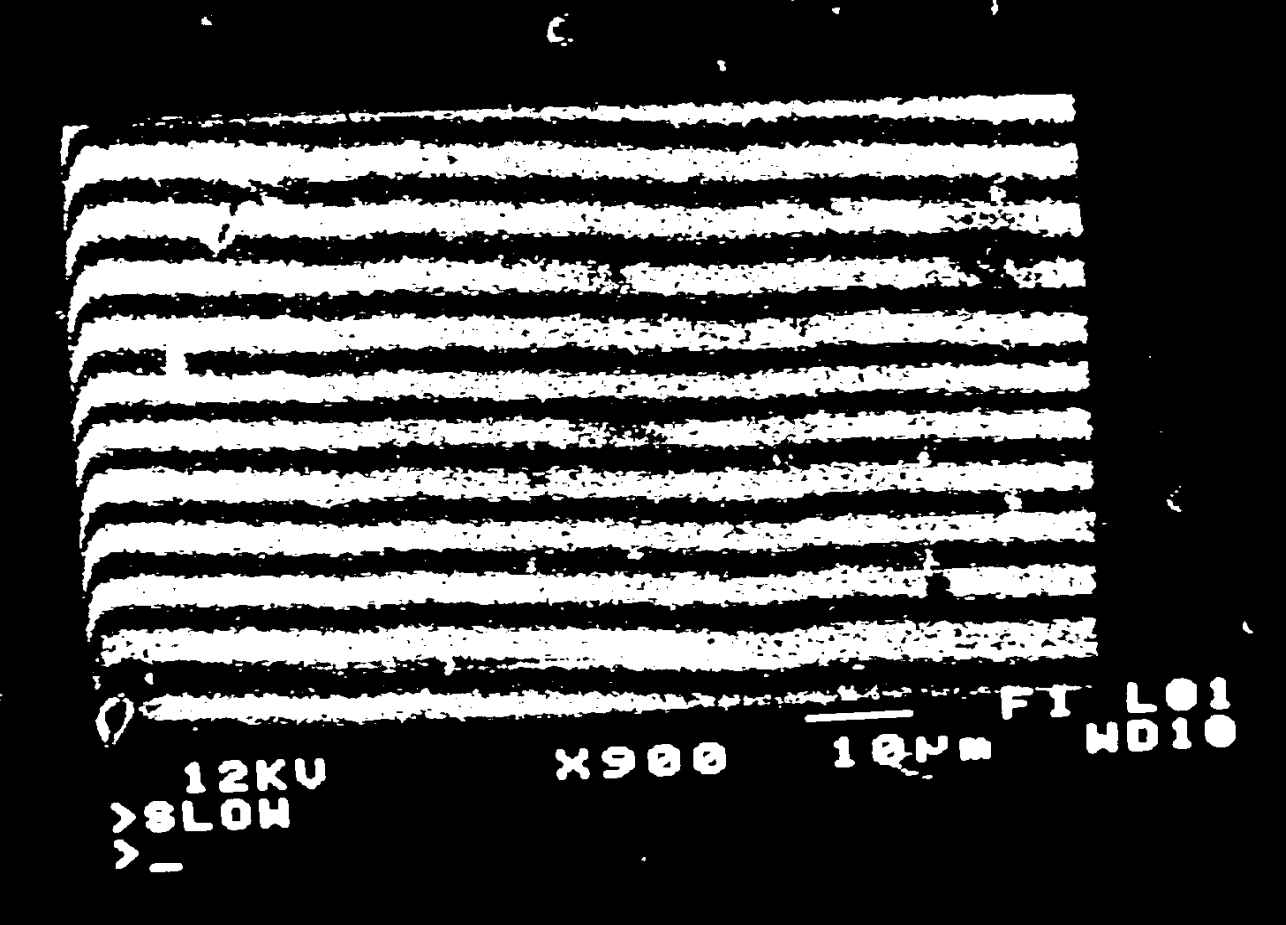
As in Fig. 4a except at a magnification of 900.

**Fig. 4c:**
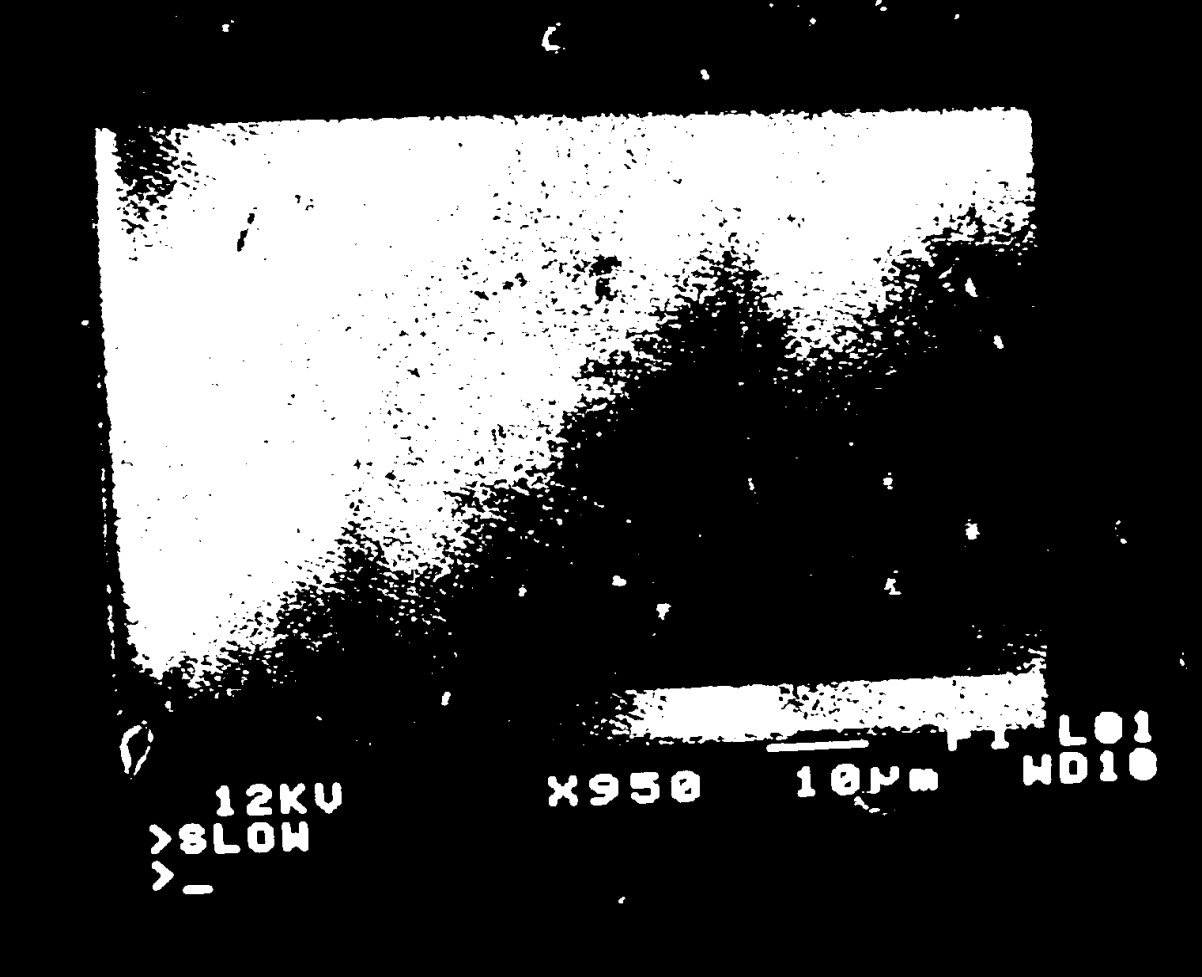
As in Fig. 4a, except at a magnification of 950.

**Fig. 4d:**
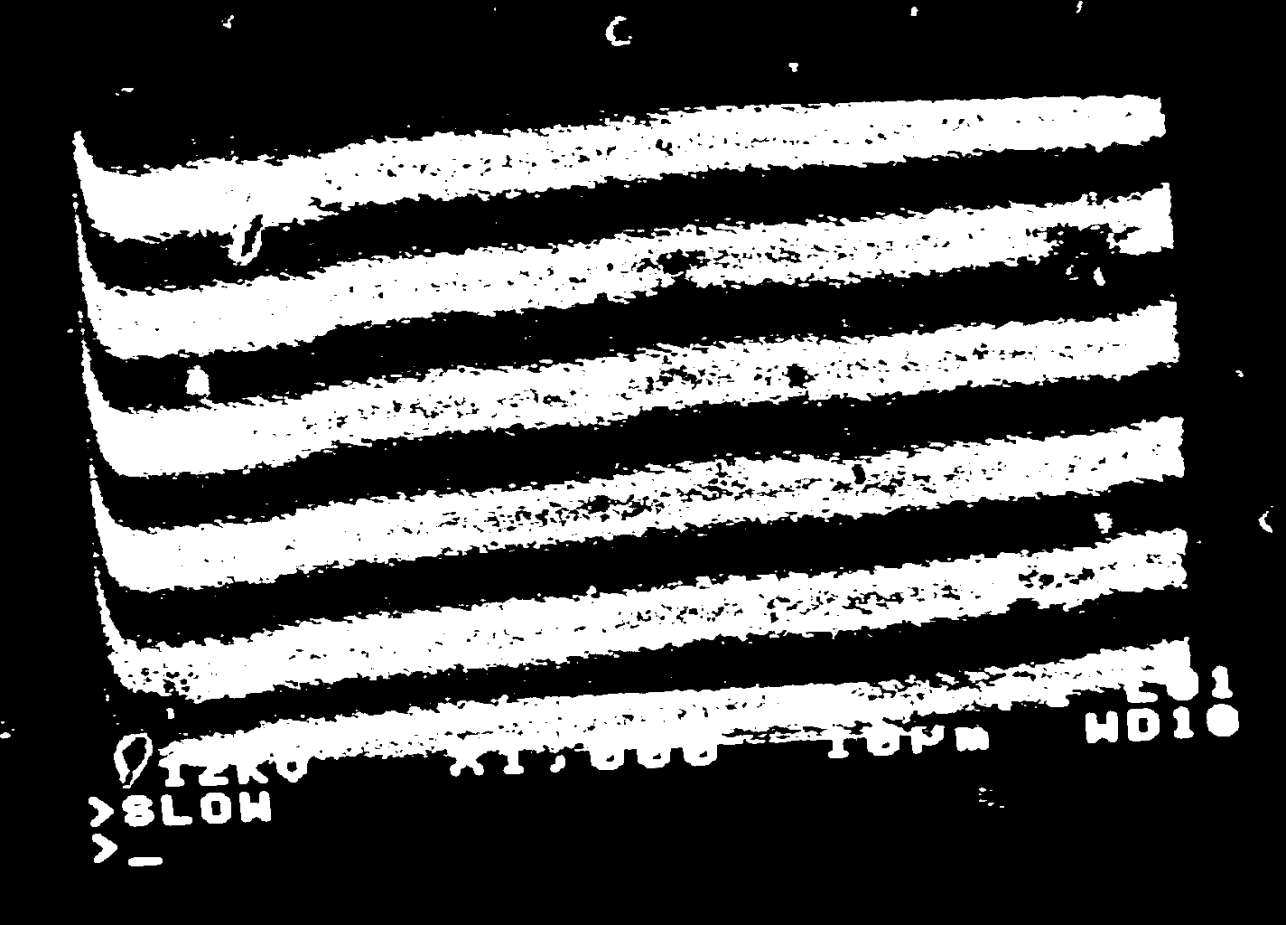
As in Fig. 4a, except at a magnification of 1000.

**Fig. 4e:**
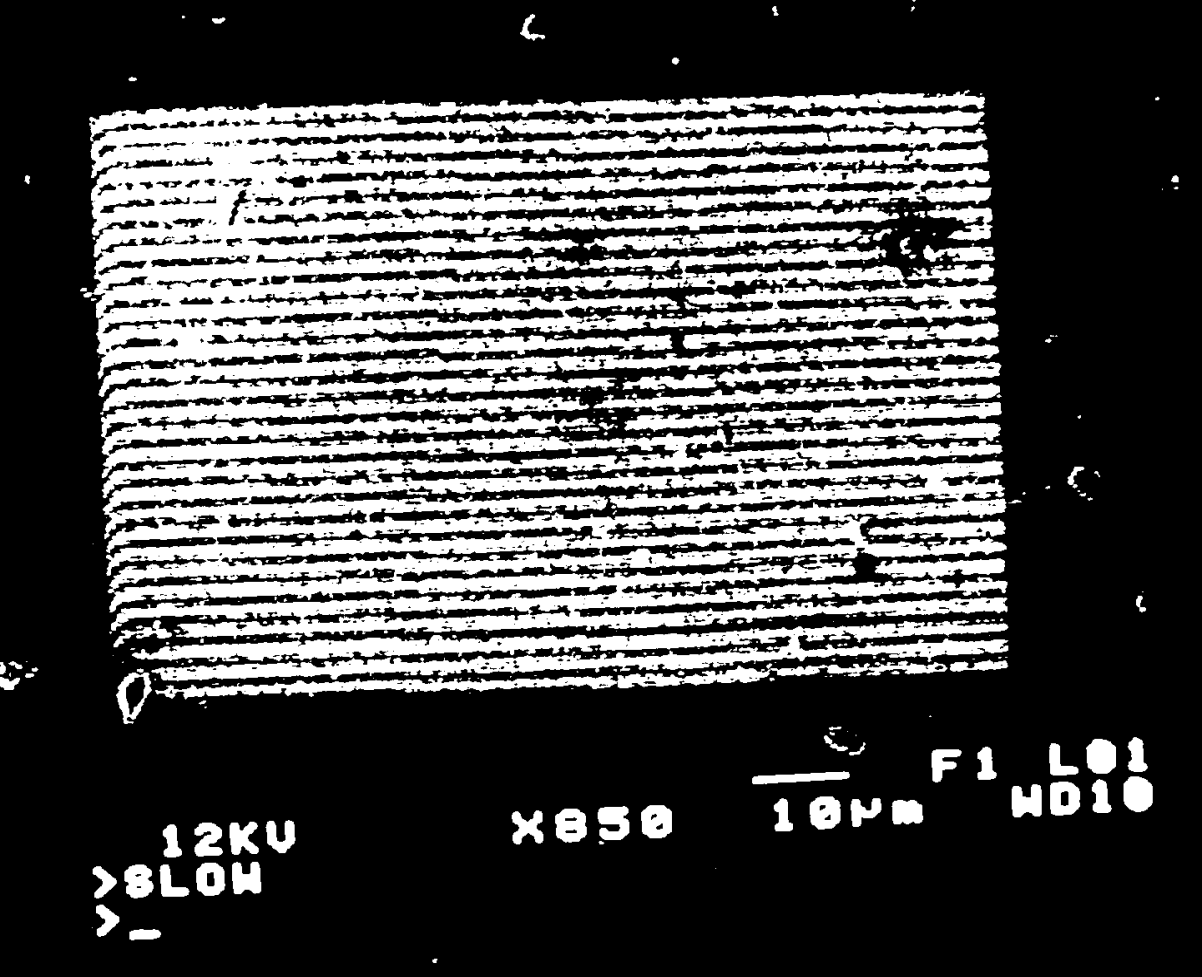
As in Fig. 4a, except at a magnification of 1100.

**Fig. 5a:**
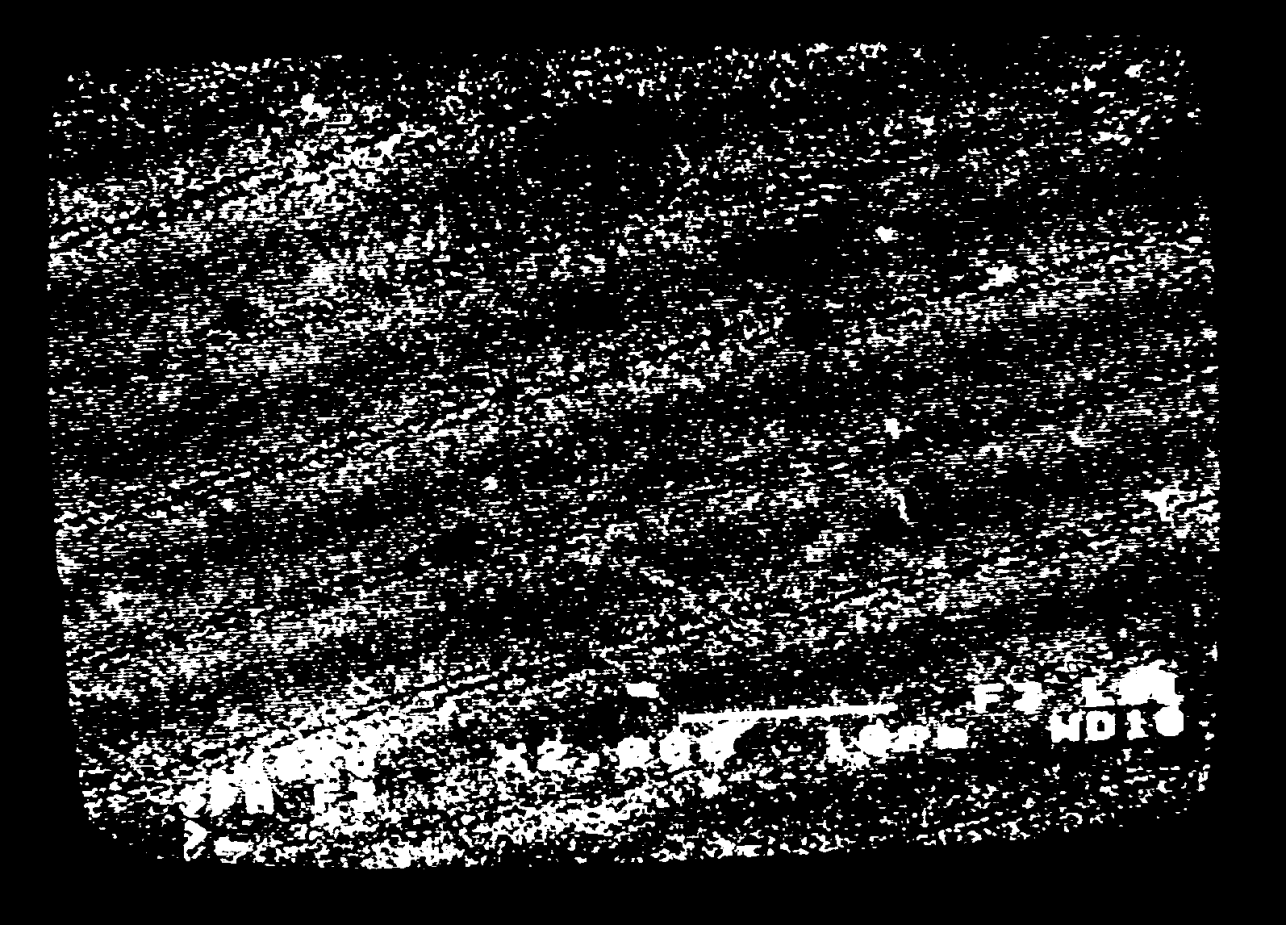
Moiré fringes of multiplication with *p*_g_ = 200 nm at a magnification of 2000.

**Fig. 5b:**
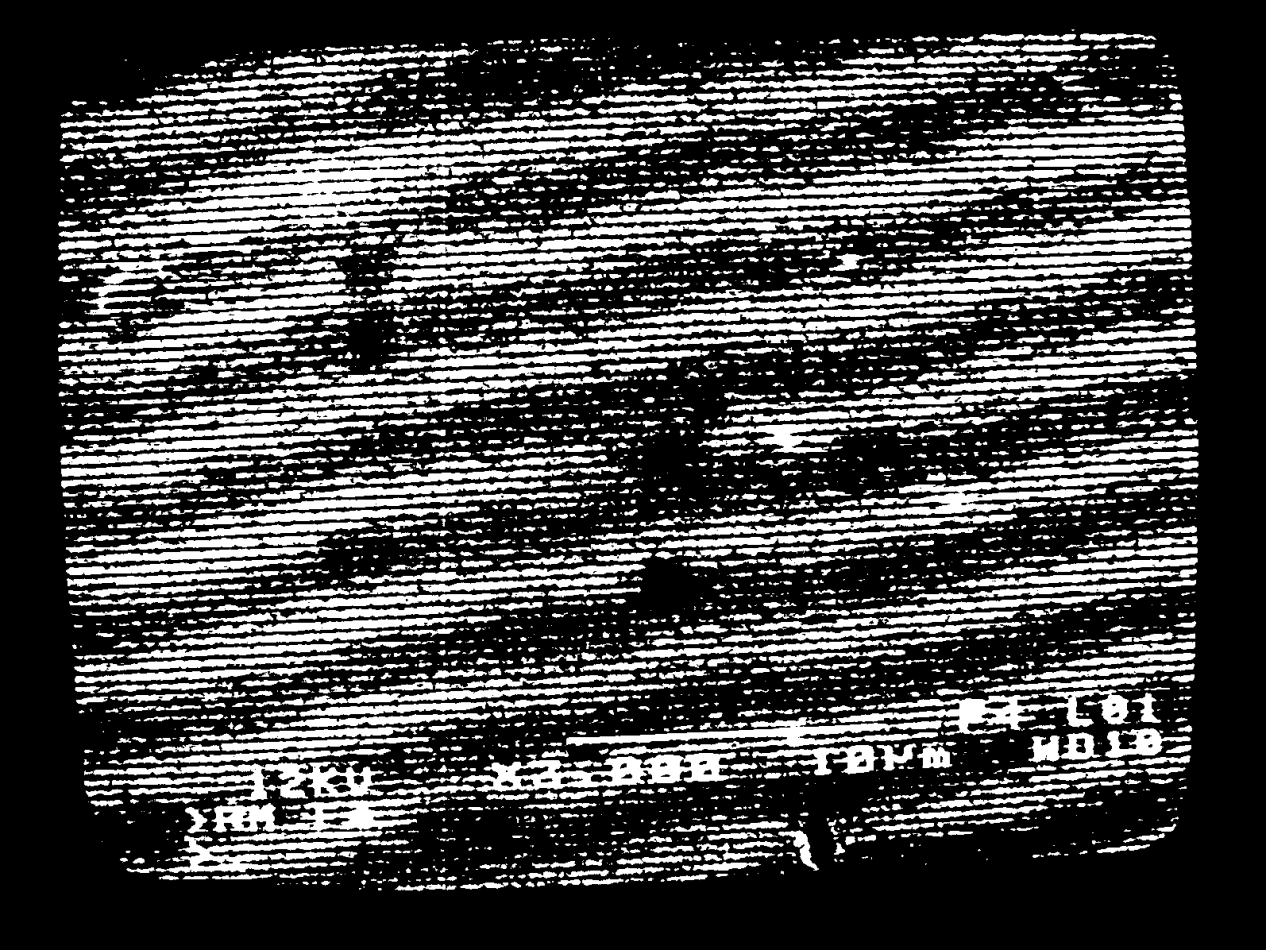
As in Fig. 5a, except at a magnification of 3000.
